# Unraveling the molecular mechanisms of nitrogenase conformational protection against oxygen in diazotrophic bacteria

**DOI:** 10.1186/1471-2164-11-S5-S7

**Published:** 2010-12-22

**Authors:** Letícia MS Lery, Mainá Bitar, Mauricio GS Costa, Shaila CS Rössle, Paulo M Bisch

**Affiliations:** 1 Instituto de Biofísica Carlos Chagas Filho, Universidade Federal do Rio de Janeiro, 21949-901, Rio de Janeiro, Brasil; 2 Department of Earth and Environmental Sciences, Center for Nanoscience, Ludwig-Maximilians-Universität München, 80333 Munich, Germany

## Abstract

**Background:**

*G. diazotrophicus* and *A. vinelandii* are aerobic nitrogen-fixing bacteria. Although oxygen is essential for the survival of these organisms, it irreversibly inhibits nitrogenase, the complex responsible for nitrogen fixation. Both microorganisms deal with this paradox through compensatory mechanisms. In *A. vinelandii* a conformational protection mechanism occurs through the interaction between the nitrogenase complex and the FeSII protein. Previous studies suggested the existence of a similar system in *G. diazotrophicus*, but the putative protein involved was not yet described. This study intends to identify the protein coding gene in the recently sequenced genome of *G. diazotrophicus* and also provide detailed structural information of nitrogenase conformational protection in both organisms.

**Results:**

Genomic analysis of *G. diazotrophicus* sequences revealed a protein coding ORF (Gdia0615) enclosing a conserved “fer2” domain, typical of the ferredoxin family and found in *A. vinelandii* FeSII. Comparative models of both FeSII and Gdia0615 disclosed a conserved beta-grasp fold. Cysteine residues that coordinate the 2[Fe-S] cluster are in conserved positions towards the metallocluster. Analysis of solvent accessible residues and electrostatic surfaces unveiled an hydrophobic dimerization interface. Dimers assembled by molecular docking presented a stable behaviour and a proper accommodation of regions possibly involved in binding of FeSII to nitrogenase throughout molecular dynamics simulations in aqueous solution. Molecular modeling of the nitrogenase complex of *G. diazotrophicus* was performed and models were compared to the crystal structure of *A. vinelandii* nitrogenase. Docking experiments of FeSII and Gdia0615 with its corresponding nitrogenase complex pointed out in both systems a putative binding site presenting shape and charge complementarities at the Fe-protein/MoFe-protein complex interface.

**Conclusions:**

The identification of the putative FeSII coding gene in *G. diazotrophicus* genome represents a large step towards the understanding of the conformational protection mechanism of nitrogenase against oxygen. In addition, this is the first study regarding the structural complementarities of FeSII-nitrogenase interactions in diazotrophic bacteria. The combination of bioinformatic tools for genome analysis, comparative protein modeling, docking calculations and molecular dynamics provided a powerful strategy for the elucidation of molecular mechanisms and structural features of FeSII-nitrogenase interaction.

## Background

Nitrogen is a component of nucleic acids, proteins and many other biological molecules thus, it is an essential element for all living organisms. Although the N_2_ gas is abundant in the atmosphere, it can not be readily used by most organisms. Nitrogen fixation is a key process in which molecular nitrogen is reduced to form ammonia, which is the form used by living systems for the synthesis of many organic compounds [1]. Biological nitrogen fixation is catalyzed by the oxygen-sensitive enzyme nitrogenase present in some microorganisms known as diazotrophs, mainly bacteria [2-4].

The most abundant and extensively studied nitrogenase is the molybdenum (Mo)-dependent enzyme [3]. It is composed of two metalloproteins: the MoFe-protein (also called dinitrogenase or component I) and the Fe-protein (also called dinitrogenase reductase or component II) [2]. Both the MoFe and Fe-protein are irreversibly damaged by oxygen [5]. O_2_ exposure leads to inappropriate oxidation of the metalloclusters, decrease of protein secondary structure and further degradation [6].

Yet, several nitrogen-fixing bacteria are aerobic and demand high O_2_ flux for proper cellular metabolism. Therefore these bacteria present mechanisms to solve this apparent paradox. Under high O_2_ concentration, they increase metabolism and consume more carbon source than needed to satisfy its energy requirement for growth in order to decrease O_2_ pressure [7,8]. Subtle adjustments in the composition and functioning of the respiratory chain, mainly differential expression of cytochromes, were described [9-11]. Additionally, diazotrophs grown in air secrete abundant extracellular polysaccharides and live inside colonies that help to produce a local microaerobic environment [12,13].

Besides the respiratory protection mechanism, it was shown that a few nitrogen-fixing bacteria present a conformational protection mechanism of nitrogenase against O_2_[14-17]. Such system involves the interaction of a ferredoxin protein (FeSII or Shethna protein) with the nitrogenase complex that results in stabilization of a reversibly inactive complex. The ferredoxin protein is found as an homodimer that contains a 2[Fe-S] cluster and interacts specifically and reversibly with the nitrogenase complex [18].

Similar mechanisms were described in *Azotobacter chroococcum*[14], *Azotobacter vinelandii*[15], *Clostridium pasteurianum*[19,20] and *Klebsiella pneumoniae*[17]. The FeSII protein was mainly studied in *A. vinelandii*[18,21,22]. Site-directed mutagenesis and crosslinking experiments revealed two residues possibly with fundamental roles on the interaction of FeSII with nitrogenase [20,23].

Previous studies have shown evidences of a similar mechanism of conformational protection in *Gluconacetobacter diazotrophicus*, however the putative FeSII protein was not yet identified [16].

Due to the great importance of biological nitrogen fixation for sustainable crop production, advances have been achieved in genetics and biochemistry, culminating in the determination of the crystallographic structures of both nitrogenase components [24-26]. However, studies on protein regulation and dynamics need to be carried out to a complete understanding of the molecular nature of the process.

In order to gain knowledge on structure and function of the proteins involved in the conformational protection of nitrogenase, we performed bioinformatic analysis on the whole genome sequence of *G. diazotrophicus* and identified a putative FeSII protein. In addition, molecular modeling, dynamics and docking studies on both *A. vinelandii* and *G. diazotrophicus* FeSII proteins and nitrogenases were carried out, elucidating molecular aspects of protein-protein interaction.

## Results and discussion

### Gdia0615 codes for a putative FeSII protein of *G. diazotrophicus*

*C. pasteurianum* and *A. vinelandii* FeSII proteins were target of several theoretical and experimental studies [15,19-23,25]. Thus, its primary sequences were used to search for an homologue in *G. diazotrophicus*. Searches were performed on both versions of *G. diazotrophicus* complete genome sequence available, containing 3852 (Riogene; [27]) and 3501 (DOE) predicted protein coding genes. However, similarity searches on predicted protein sequences or raw genomic sequence did not return any hit with high sequence similarity (>30% identity) and coverage (>70%).

In order to follow up the search for a putative FeSII in *G. diazotrophicus* the functional properties of FeSII proteins were analyzed. For instance, *A. vinelandii* FeSII protein presents 13 kDa and a fer2 domain [21], found in ferredoxins that are electron carrier proteins with a 2Fe-2S cofactor acting in a wide variety of metabolic reactions. The members of this family are proteins of approximately one hundred aminoacids with four conserved cysteine residues that coordinate the 2[Fe-S] cluster and have a general core structure consisting of beta(2)-alpha-beta(2) [28].

Accordingly, Ureta et al, 2002 [16] have shown the co-precipitation of a 14 kDa protein with nitrogenase of *G. diazotrophicus* cells grown diazotrophically under high oxygen concentration. This result suggested a conformational protection mechanism of nitrogenase in *G. diazotrophicus*, involving a 14 kDa protein.

In a second attempt to identify the *G. diazotrophicus* FeSII protein, we have analyzed the whole genome sequences for ferredoxin proteins. From the 7353 ORFs analyzed, a search for textual annotation with the “ferredoxin” or “2Fe-2S” syntax revealed 16 putative ferredoxins. From these, only 8 present predicted molecular weight between 9 and 20 kDa, around the expected size for a putative 2[Fe-S] Shethna protein. Analysis of these 8 ferredoxins for the presence of functional domains disclosed two ORFs in *G. diazotrophicus* genome with high score for the fer2 domain (Protein Family PF00111; Gdi2370 and Gdia0615 both presented an e-value of 7.2e^-17^). However, they correspond to the same protein, as they come from one of each complete genome sequence available. They are 99% identical and the only difference is that Gdia0615 is 3 aminoacids longer (initial MPH sequence) than Gdi2370. Thus, Gdia0615 was chosen for further analysis.

**Figure 1:**
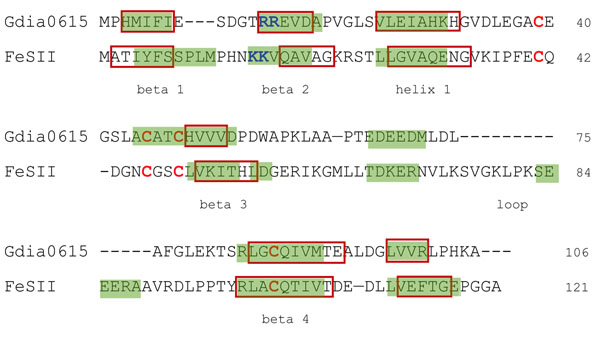
**Alignment of primary sequences of *A. vinelandii* FeSII and Gdia0615 (putative *G. diazotrophicus* FeSII protein)** The predicted beta-sheets and alpha-helices are shaded in green . Beta-sheets and alpha-helices present in the comparative models are indicated inside red boxes. Conserved lysine and arginine residues (K and R), important for FeSII interaction with nitrogenase complex are indicated in blue. Cysteines involved in 2[Fe-S] cluster coordination are shown in red.

In order to assert the putative function of Gdia0615, prediction of secondary structure of *A. vinelandii* FeSII was compared to the predicted secondary structure of Gdia0615. Both sequences form 2 beta sheets followed by an alpha helix and 2 subsequent beta-sheets (Fig. 1). *A. vinelandii* FeSII presents a 13 residues larger loop in the region 77-90. In summary, the secondary structure profile of both proteins is highly similar and the only structure discrepancy is the mentioned loop region (Fig. 1).

Following this analysis, the functional conserved residues from *A. vinelandii* FeSII were investigated for corresponding residues on the putative *G. diazotrophicus* sequence. Lou et al 1999 [23] described the importance of two lysine residues (K14 and K15) crucial for FeSII interaction with nitrogenase. Interestingly, Gdia0615 presents two arginine residues (R13 and R14) in a conserved position (Fig. 1). K and R are both basic residues and might encompass same function in the protein context. Besides, *A. vinelandii* FeSII presents 4 cysteine residues at positions 42, 47, 50 and 102, that coordinate the metal cluster, essential for plenty function of FeSII [23]. Gdia0615 also presents this 4 cysteine residues well conserved in structure (C39, C45, C48 and C85; Fig. 1). Altogether, these results strongly suggest that Gdia0615 is the gene that codes for the putative FeSII in *G. diazotrophicus*.

### FeSII molecular models revealed a conserved β-grasp core structure

The results discussed above strongly suggest that Gdia0615 is the putative FeSII protein of *G. diazotrophicus*. However, there are no structural information available for both *A. vinelandii* FeSII and Gdia0615. It is far known that protein structure is a determinant of protein function and three-dimensional structure is more conserved than protein sequence. Thus, as a support to the above predictions, 3D comparative models for Gdia0615, as well as for *A. vinelandii* FeSII, were constructed.

The first step in comparative modeling is the definition of a template sequence. The template sequence must have its 3D structure experimentally determined with high accuracy and present sequence similarity with the target. The quality of 3D structure (resolution of the template structure) and the alignment between template and target sequences are crucial for the model quality. Therefore, several alignments were generated for the FeSII sequences, using information from sequence database searches, secondary structure prediction and available homologues with resolved 3D structures. Alignments were then manually optimized in order to minimize gaps and assert that the conserved domain residues are aligned. Although Gdia0615 and *A. vinelendii* FeSII might both perform the conformational protection of nitrogenase, they share low sequence similarity (18% identity and 42% similarity). Therefore, template structures chosen for comparative modeling of each of them were different. Gdia0615 chosen template is FdvI protein (48% identity and 67% similarity), a 2[Fe-S] ferredoxin essential for growth of *Rhodobacter capsulatus*[28], also a diazotrophic bacterium. *A. vinelandii* FeSII chosen template is *E. coli* Fdx (20% identity and 36% similarity), an adrenodoxin-type 2[Fe-S] ferredoxin with an essential role in the maturation of various iron-sulfur (Fe-S) proteins [29]. This two template proteins share some structural similarity and both coordinate a 2[Fe-S] cluster.

The *A. vinelandii* FeSII model (deposited at Protein Model DataBase at identification number PM0075978) exhibited high stereochemical quality (97% of residues in the allowed regions of Ramachandran plot) and a high probability to represent a native-like conformation (Dope-score -3.7493 and ProSa Z-score -5.06). The average distance deviation between backbone equivalent atoms (RMSD - Root Mean Square Deviation) measures the similarity between 3D structures after optimal superposition; the RMSD score for FeSII proposed model vs. the crystal structure of Fdx was 0.53 Å, a further indication of the high quality of the model [30]. A remarkable feature of this model is a large loop region, comprising aminoacids 77-90, as expected from the observed gap in sequence alignment. In overall, this represents a structure of high quality.

The Gdia0615 model (deposited at PMDB at identification number PM0075980) also presented high stereochemical quality (100% of residues in the allowed regions of Ramachandran plot) and is a highly probable native-like structure (Dope-score -4.0447 and ProSa Z-score -6.57). The RMSD between Gdia0615 model and FdvI crystal structure is 0.46 Å, representing the overall quality of the model.

**Figure 2:**
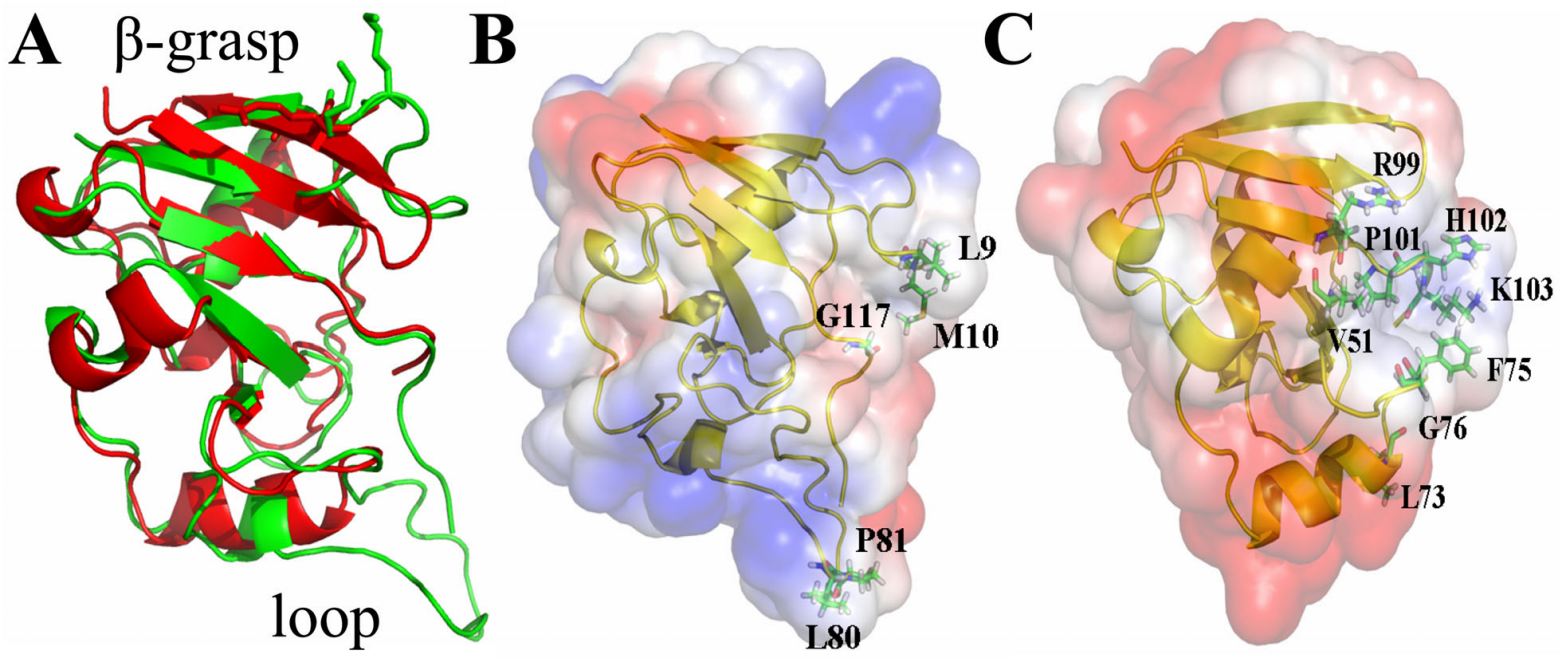
**The β-grasp fold and electrostatic surface of molecular models of FeSII and Gdia0615** The β-grasp structure is shown in yellow. *A. vinelandii* FeSII (green) fitted to *G. diazotrophicus* Gdia0615 (putative FeSII; red) is shown in panel A. Electrostatic surface of FeSII (B) and Gdia0615 (C) 3D molecular models are also represented. Blue indicates positive charged residues, red represents negative areas and white are neutral regions. Active residues for docking calculations are numbered.

In overall, the molecular models obtained are highly informative since their templates present high resolution and molecular modeling included alignment optimization, loops refinement, secondary structure prediction and metalloclusters positioning. Although FeSII and Gdia0615 models were built from different template structures, they share many structural features. The RMSD between their structures is only 1.46 Å (Fig. 2A). This reflects the protein structure conservation, the most required feature for proper protein function maintenance. Both models present 4 well defined beta-sheets and 1 structural alpha-helix (Fig. 1). The specific arrangement of these structural elements is a conserved motif typical of the general core structure characteristic of ferredoxin proteins, known as beta-grasp fold (Fig. 2). The beta-grasp fold supplies an effective scaffold for binding iron-sulfur clusters in the case of the 2Fe-2S ferrodoxins [31].

Additionally, the 4 cysteine residues are positioned towards the 2[Fe-S] cluster, as was expected, in both models (Fig. 2). The 2 lysine (K14 and K15) residues of *A. vinelandii* FeSII and the corresponding 2 arginine (R13 and R14) of *G. diazotrophicus* are positioned at exposed surface and might be involved in nitrogenase interaction, as suggested before.

Altogether these results provide evidence that FeSII and Gdia0615 could perform same molecular function.

### FeSII dimer is the stable functional unit

**Figure 3:**
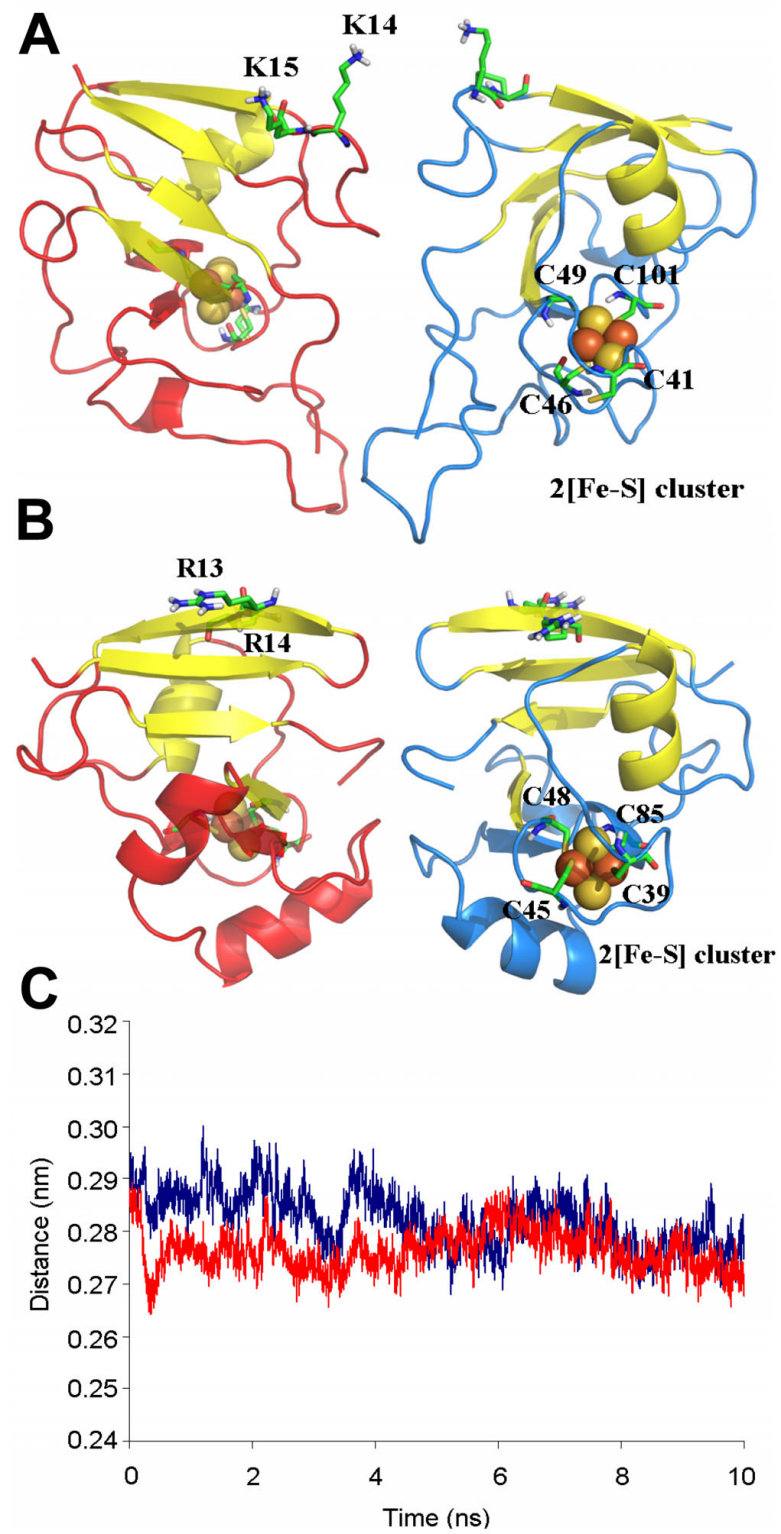
**Conserved core structure of the dimer complexes of FeSII and Gdia0615 are stable** A) *A. vinelandii* FeSII dimer. B) *G. diazotrophicus* Gdia0615 (putative FeSII) dimer. C) Distance of center of mass between each subunit of *A. vinelandii* FeSII (blue) or *G. diazotrophicus* Gdia0615 (red) throughout 10ns of molecular dynamics simulations

Previous studies detected that *C. pasteurianum* and *A. vinelandii* FeSII binds to nitrogenase complex as an homodimer. Analyses of electrostatic potential and solvent exposed surface of the monomer molecular models were performed to identify a possible dimerization interface. Electrostatic potential of *A. vinelandii* FeSII revealed a predominant positive structure. However, protein-protein dimerization surfaces are typically neutral, as hydrophobic residues are usually found enclosed inside the complex structure. Charged residues largely exposed at protein surface may also contribute to protein-protein interactions through salt bridges and hydrogen bonds. Careful analysis of solvent accessible residues showed a possible region of dimerization comprising hydrophobic and basic residues accessible to solvent comprising L9, M10, G116, L80 and P81. Therefore, these residues were set as obligatory contacts for docking calculations. Additionally, the large loop region (residues 77-94) presents some hydrophobic aminoacids and consequently was set as a semi-flexible region for docking experiments. The resulting dimer (Fig. 3) presented high quality (ProSa Z-score -6.08), a symmetric structure (each monomer is horizontally rotated in approximately 180° in relation to the other) and complementarities of shape and charge in the dimerization interfaces. The loop region seems to strengthen protein dimerization. A 10 ns molecular dynamics simulation in aqueous solution revealed that the dimer is stable (RMSD = 3.08 Å; standard deviation = 0.041 Å), supporting the hypothesis that the dimer is the functional unit. Gdia0615 showed a similar pattern of charge distribution in comparison to FeSII (Fig. 2). Mainly positive residues are found in the surface. From the solvent exposed surface residues, the most notable ones (V51, L73, F75, G76, R99, P101, H102 and K103) were selected as obligatory contacts for docking experiment. Calculations resulted in dimer structure with native-like structure (ProSa Z-score -4.52) that close resembles the *A. vinelandii* FeSII dimer model (Fig. 3). Shape and charge complementarities of the surface interface of the proposed dimer are according to physicochemical restrictions. Gdia0615 dimeric orientation is very similar to FeSII (Fig. 3) and showed stable behavior after 10 ns of dynamics simulation (RMSD = 2.45 A° +- 0.043 A°).

### *G. diazotrophicus* nitrogenase complex structure is close related to *A. vinelandii* nitrogenase

In order to better understand the conformational protection mechanism of nitrogenase from oxygen damage, analysis of FeSII-nitrogenase interaction is required. The protection is due to the formation of an oxygen-tolerant three-component nitrogenase complex (Fe-protein + MoFe-protein + FeSII protein) [16,32]. Crystal structures of *A. vinelandii* nitrogenase complex were already determined and presented insights on multiple docking sites of nucleotides [33,34]. Mutant forms of nitrogenase proteins were also studied and revealed key residues for nucleotide-protein interaction [24]. In contrast, FeSII interaction with nitrogenase complex was not yet studied in respect to structural aspects. In addition, it lacks information on 3D structure of *G. diazotrophicus* nitrogenase complex.

As a strategy to fulfill this gap, a molecular model of *G. diazotrophicus* nitrogenase complex was built through comparative modeling. The Fe-protein presents 70% identity and 85% positivity with the *A. vinelandii* homologue. The MoFe-protein α-subunit shows 71% identity and 81% similarity with the *A. vinelandii* counterpart. The β-subunit of MoFe-protein is 52% identical and 70% similar to the *A. vinelandii* protein. In summary, all components of nitrogenase complex from *G. diazotrophicus* and *A. vinelandii* share high sequence similarity. Therefore, the template structures for both components of nitrogenase complex were the *A. vinelandii* subunits.

In consequence of this high degree of similarity between target and template sequences, the molecular model of *G. diazotrophicus* nitrogenase complex presented high stereochemical quality. Molecular models of Fe-protein (deposited at PMDB at identification number PM0075981), MoFe-protein α and β-subunits (deposited at PMDB at identification numbers PM0075982 and PM0075983, respectively) presented 99%, 98% and 99% of residues in allowed regions of Ramachandran plot. The overall RMSD between each crystal structure and the proposed model is 0.41, 0.31 and 0.54 Å, respectively. Such a high quality model is suitable for protein-protein interaction analysis.

### A negative pocket anchors the FeSII positive region near the Fe-protein/MoFe-protein interface in the nitrogenase complex

As mentioned before, a few studies reported the interaction of FeSII with nitrogenase. Nitrogenase protein is found as an oligomer (two Fe-protein homodimers + one heterotetramer of MoFe-protein). The binding site of FeSII in nitrogenase is not clearly described. It was shown that a 2[Fe-S] ferredoxin of *C. pasteurianum* binds the MoFe-protein at a site involving both subunits of the MoFe-protein [35]. However, in *A. vinelandii* and *A. chroococcum* it was demonstrated that FeSII binds to the nitrogenase complex, but not to each individual subunit [23,32,36]. Apparently, the formation of a three-component nitrogenase complex (Fe-protein + MoFe-protein + FeSII) is essential for nitrogenase oxygen tolerance and suggests that FeSII interacts near the subunits interface of nitrogenase complex [32,36]. Furthermore, in *A. vinelandii*, several site-directed mutants were constructed and tested for its FeSII capability of interaction with nitrogenase [23]. Two of those (K14 and K15) lost ability of interaction. This experiment showed the importance of these two K residues for interaction. However, it was not shown if is due to direct contact or allosteric effect.

**Figure 4:**
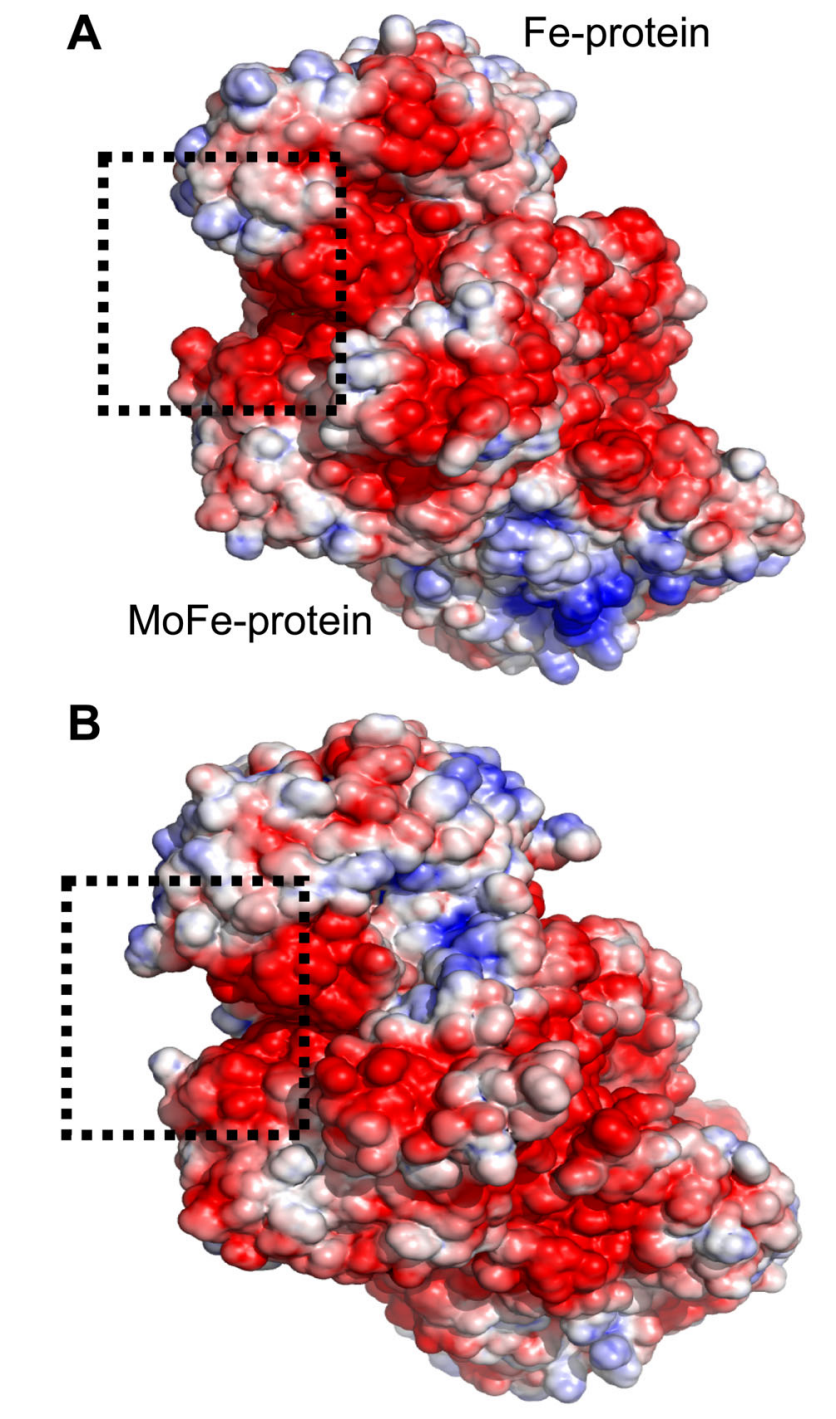
**Electrostatic surface of nitrogenase complex of *A.vinelandii* and *G.diazotrophicus*** The positive regions are indicated in blue and the negative regions in red. The squares indicate the interaction site of FeSII. A) *A. vinelandii* nitrogenase complex. B) *G. diazotrophicus* nitrogenase complex.

The oxygen-stable three-component complex is very large to perform blind docking calculations in a feasible computational time. In order to elucidate this mechanism, electrostatic surface, shape and physicochemical complementarities of both FeSII and nitrogenase complex were exhaustively inspected. *A. vinelandii* Fe-protein presented 3 glutamic acid residues (E69, E112 and E113) at Fe-protein surface near the interface with MoFe-protein. This negative area visually fits in shape and charge the FeSII region around K14 and K15, the two lysine that may have an important role in the initial steps of recognizing the nitrogenase component residues (Fig. 4; [23]). In addition, the histidine 55 (H55) is in this same structural interface. It was suggested that H55 might modulate the FeSII protein’s affinity for nitrogenase in a redox state-dependent manner [23].

*G. diazotrophicus* Gdia0615-nitrogenase interaction was also analyzed. The surface electrostatic potential and structural shape of both were manually inspected. As observed in *A. vinelandii* proteins, there is a small negative pocket on the interface of Fe-protein and MoFe-protein. This region is completely complementary in shape and charge to the unique positive region of Gdia0615, corresponding to R13 and R14 neighborhood. Such suspicious analysis suggests these arginine residues are directly involved in the nitrogenase oxygen-stable three-component complex formation in *G. diazotrophicus* (Fig. 4).

In order to support these analyses, docking calculations of *A. vinalendii* FeSII protein and nitrogenase complex were performed. According to the evidences described above, nitrogenase E69, E112 and E113 residues and FeSII K14, K15 and H55 were set as active residues for docking calculation (Table 1).

**Table 1:** FeSII and nitrogenase interacting residues

FeSII	Nitrogenase	Distance (A°)	Type
**K14**	E71*	**6.5**	**Salt bridge**

**K14**	E73*	**2.5**	**H-bond (side chain-side chain)**

**K15**	D74*	**6.6**	**Salt bridge**

**K15**	E73*	**4.6**	**Salt bridge**

**K15**	K84*	**3.8**	**H-bond (side chain-backbone)**

**Y4'**	E288*	**4.0**	**H-bond (backbone-side chain)**

**K14'**	E112*	**5.9**	**Salt bridge**

**K15'**	**E288**	**2.7**	**H-bond (side chain-side chain)**

**Q17'**	**E287**	**2.6**	**H-bond (side chain-backbone)**

**K36'**	**D388**	**3.2**	**H-bond (side chain-side chain)**

FeSII β-chain are indicated by an (’). Nitrogenase residues marked with an asterisk (*) belong to the Fe-protein and those without are from MoFe-protein. Polar interactions were classified in salt bridges and hydrogen bonds. Atoms participating on these contacts were separated in backbone or side-chain atoms.

From the 1000 complexes generated, 49 are clustered in the same docking site and show a very similar conformational structure. They also represent the lowest energy complexes obtained and the best one is shown on figure 4 (Haddock score = -59.8 with standard deviation of 2.6). The complex structure presented energy values of: -763.5 +/- 41.8 (electrostatic contribution), -26.8 +/- 6.3 (van de Waals), 102.8 +/- 12.4 (desolvation energy) and 169.4 +/- 47.81 (restraints penalty energy). Analysis of these energetic components of FeSII-nitrogenase complex confirmed that interaction is mainly due to electrostatic potential. This result explains previous experimental data which shows that an increase in ionic strength disrupts the FeSII-nitrogenase complex [32,36].

**Figure 5:**
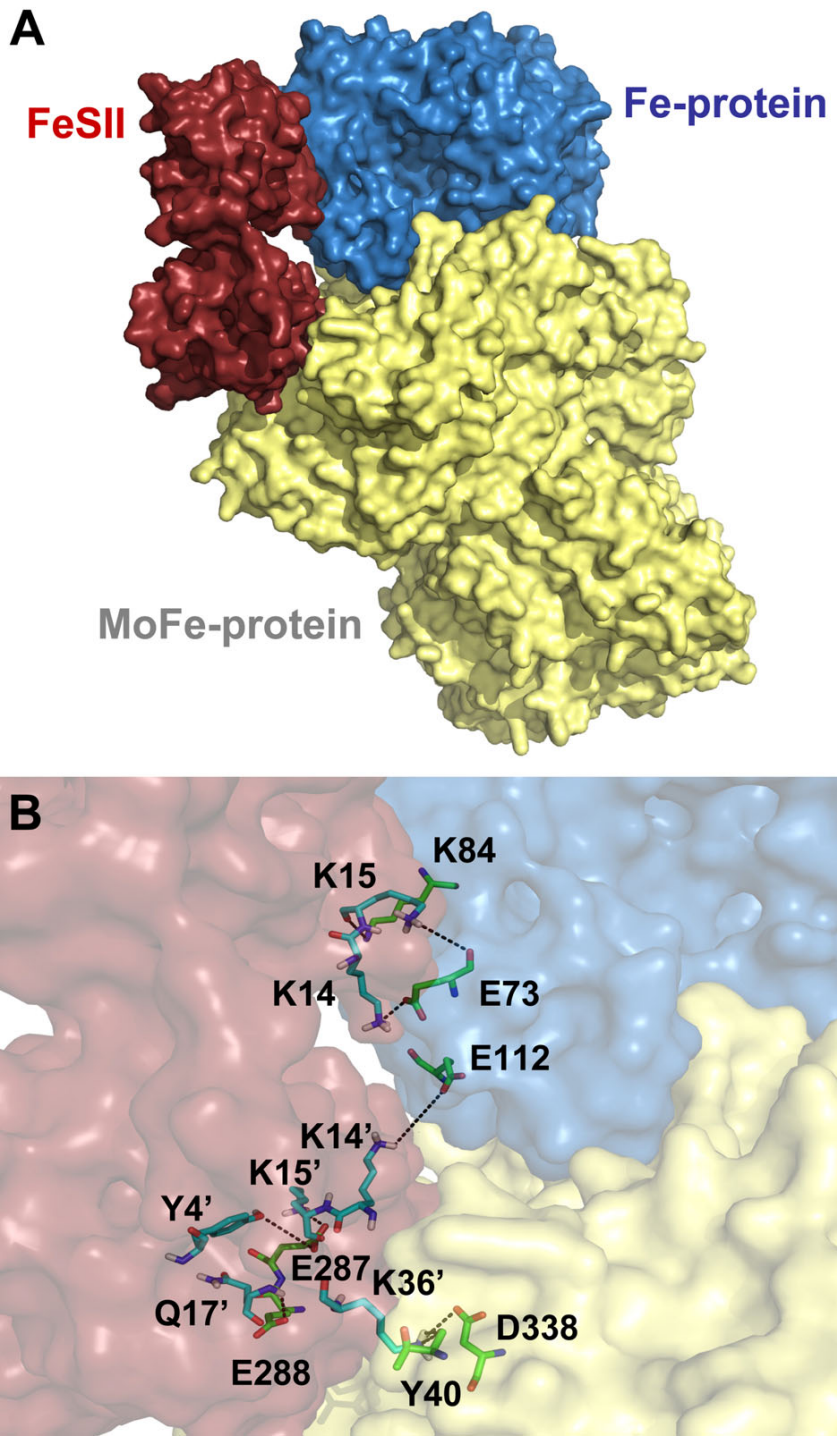
**The oxygen stable three-component complex of *A. vinelandii*** A) Surface representation of FeSII (red), Fe-protein (blue) and MoFe-protein (yellow). B) A closer view of the interaction interface. Residues involved in interaction are highlighted.

Further, the docking complex structure revealed that FeSII K14 and K15 are directly involved in the complex formation, supporting the earlier mutagenesis data [23]. Fe-protein residues engaged on interaction are E68, E71, E73, D74 and E112. MoFe-protein main aminoacids are E288 and D385 (Fig. 5). Additional residues also participate on molecular interactions, either through hydrogen bonds, saline contacts or electrostatic interaction (Tab. 1).

## Conclusions

The nitrogenase conformational protection mechanism against oxygen is a bacterial strategy to avoid the enzyme degradation during a sudden increase in oxygen concentration. FeSII protein (Shethna protein) interacts with nitrogenase complex [19,21]. The three-component complex keeps nitrogenase inactive but transiently protected from damage by oxygen [15]. This oxygen-stable nitrogenase complex is formed only under conditions of low ionic strength in the presence of MgCl_2_, and requires all three components to be present in an oxidized state [32,36].

*A. vinelandii* FeSII was target of a few studies regarding its function and regulation. This study contributed for the understanding of this mechanism with structural aspects. In contrast, *G. diazotrophicus* protein involved in nitrogenase conformational protection was not yet known. The results described herein present for the first time a putative FeSII protein for *G. diazotrophicus*. A detailed analysis of all predicted ORFs of complete genomic sequence revealed only one possible protein coding gene (Gdia0615) for the putative FeSII. Although Gdia0615 present low sequence similarity with *A. vinelandii* FeSII, all functional residues are well conserved. A comparative analysis of the 3D molecular models of Gdia0615 and FeSII support the hypothesis of Gdia0615 protective function. Both form a beta-grasp folding and present similar electrostatic properties. In addition, they are functional as symmetric homodimers that interact through a hydrophobic interface.

*A. vinelandii* FeSII docking with nitrogenase complex revealed a putative binding site near the Fe-protein/MoFe-protein interface, corroborating previous data on the three-component complex. This interaction is dependent of shape and charge complementarities. Two FeSII lysine residues (K14 and K15), as well as an histidine H55 participate actively on interaction. On the other hand, 3 glutamic acid residues of Fe-protein enclose a negative cavity for FeSII binding. In *G. diazotrophicus* we suggest that arginine residues R13 and R14, histidine H56 and adjacent regions compose the main region for Gdia0615 interaction with nitrogenase complex. In overall, this study provided the first molecular insights on structural properties of the conformational protection mechanism of nitrogenase against oxygen. Such study will certainly contribute to a better understanding of the biological nitrogen fixation process.

## Methods

### Genomic analysis

*A. vinelandii* FeSII (gi: 451865) and *C. pasteurianum* FeSII (gi: 119942) sequences are available. These sequences were used to scan the two public available complete genome sequences of *G. diazotrophicus* (gi: 162145846 and 209542188) for a putative FeSII protein. Both genome sequences present essentially the same genes. Therefore, most of the genes in one version have a matching in the other. Sequence similarity searches were performed using BLAST algorithms [37], for both aminoacids and nucleotides. Analysis of functional domains was performed in NCBI Conserved Domains (version 2.17; 31608 PSSMs; [38]) and Pfam databases (version 23.0; 10340 families; [39]). Protein secondary structure prediction based on position-specific scoring matrices was performed on PSI-PRED server [40].

### Comparative modeling

The 3D molecular models of the FeSII (*A. vinelandii),* the putative FeSII, Fe-protein and MoFe-protein (*G. diazotrophicus*) were built by comparative modeling. The search for candidate template sequences was performed at the PDB database [41]. Templates used for modeling were the *Escherichia coli* Fdx, (1I7H), the *Rhodobacter capsulatus* FdvI (1E9M) and *A. vinelandii* nitrogenase complex (1G20), respectively. Templates and target sequences were aligned using Promals3D [42] and manually optimized with the support of the DNATagger [43] by monitoring the alignment of cysteine residues and conservation of physicochemical properties of aligned residues. Molecular models were generated using the program Modeller (version 9.7; [44]), considering the presence of heteroatoms (2 [Fe-S] cluster) if suitable. Additionally, loop refinement of *A. vinelandii* FeSII loop region, comprising the residues 77 to 90 was performed. 100 candidate models were generated for each protein system and all of them were evaluated using stereochemical quality Ramachandran plots generated by Procheck (version 3.5.4; [45]) and energy values according to Modeller Dope-score [46] and Prosa (ProSa 2003; [47]). RMSD calculations, visualization and manipulation of molecular images were performed with Pymol (version 1.2; [48]).

### Docking

Prediction of solvent accessible surface area (Naccess version 2.1.1; [49]) and evaluation of the electrostatic potential on the protein surface (APBS software package; [50]) were accounted to determine potential protein-protein binding sites. These results, together with literature information, were introduced as restraints to drive docking calculations with Haddock [51]. The Haddock advanced guru interface was used in order to support the setting of active and passive residues. A list comprising the residues predicted by Naccess as directly involved in the interaction (active residues) and the surrounding residues within a radius of 6.5 angstrons around the active residues was defined. All additional parameters were used as default values. A set of probable complexes were generated and the results were analyzed through stereochemical, cluster-sizes and energy evaluations. A thousand structures were used for rigid body docking and 5 minimisation trials. Water was used as the solvent for the last iteration and 200 structures were selected for explicit solvent refinement. A 7.5 angstrons RMSD cutoff was set for clustering of at least 4 structures. A total of 200 structures were analyzed as final results from Haddock.

### Molecular dynamics

Molecular dynamics (MD) simulations, energy minimization and trajectory analyses were carried out with GROMACS 4.05 package [52], using GROMOS96 (G53a6) force field [53]. Explicit SPC water molecules [54] were used in all simulations, in which a 14 Å layer of water molecules were added around the solute molecules, within a cubic water box, using periodic boundary conditions. Counter ions were inserted for system neutralization. LINCS [55] and SETTLE [56] were applied to constraint solute and solvent bond, respectively. Temperature was kept at 298 K by rescaling velocities with a stochastic term [57] and pressure at 1 atm using the Berendsen approach [58]. Electrostatic interactions were corrected with PME method [59], using non-bonded cutoffs of 1.0 nm for Coulomb and 1.2 nm for van der Waals. MD integration time was 2 fs.

A 3-steps energy minimization protocol was used to avoid artifacts in atomic trajectories due to conversion of potential into kinetic energies: firstly, applying the steepest-descent algorithm: *i.* 5000 steps with solute heavy atom positions restrained to their initial positions using an harmonic constant of 1 kJ/mol.nm in each Cartesian direction, allowing free water and hydrogen movements; and *ii.* 5000 steps with all atoms free to move. Subsequently, the conjugated gradient algorithm was applied for further energy minimization until an energy gradient of 42 KJ/mol.nm. A preliminary MD (1 ns), with heavy atom positions restrained, was performed for achieving solvent equilibration and system heating until 298 K. In this step, the initial velocities were generated once for each simulation. Then we performed a 10 ns production MD.

## Competing interests

The authors declare that they have no competing interests.

## Authors' contributions

LMLS designed the study, participated on genomic analysis, comparative modeling and docking and drafted the manuscript. MB designed the study and carried out the genomic analysis, comparative modeling and docking. MGSC designed the study and carried out comparative modeling, docking and molecular dynamics. SCSR conceived the study. PMB designed and coordinated the study.
